# How to Understand the Role of Insurance Mechanism in a Global Pandemic?

**DOI:** 10.3390/ijerph18136743

**Published:** 2021-06-23

**Authors:** Feng Kong

**Affiliations:** 1 College of Humanities and Development Studies, China Agricultural University, Beijing 100083, China; kongfeng0824@cau.edu.cn; 2 Center for Crisis Management Research, Tsinghua University, Beijing 100084, China

**Keywords:** global pandemic, risk transfer, crisis management, catastrophe risk, insurance mechanism, COVID-19, catastrophe risk management

## Abstract

The COVID-19 epidemic has seriously affected global economic and social development. The extent to which insurance can play a role in preventing and transferring the risk of infectious diseases has become one of the major concerns of the community. This paper first analyzes the main contents of the U.S. Pandemic Risk Insurance Act during the COVID-19 epidemic and its insights to the global audiences. Then, on the basis of the definition of global pandemic, this paper analyzes the great challenges faced by the insurability of the infectious diseases’ catastrophe from the regional impact, risk accumulation, correlation with capital markets, and accuracy of catastrophe model, and the insurability of local infectious diseases. Finally, this paper presents the key points of the top-level design of the risk transfer mechanism of infectious disease insurance in China. This paper is informative in understanding the role of insurance in the risk transfer of infectious diseases.

## 1. Introduction

The global pandemic of COVID-19 has brought a great impact on global social and economic development [1,2,3]. Therefore, the need for infectious disease insurance is urgent. The infectious disease insurance discussed in this article refers to commercial insurance rather than individual or health insurance.

In some U.S. states, such as New Jersey, some voices of the local legislature have argued that the insurance mechanism should cover the financial losses of small and medium-sized businesses shutting down as a result of the COVID-19 outbreak, even hoping to break the limits of the current insurance and reinsurance provisions for the infectious diseases’ coverage. However, New Jersey’s proposal was suspended by the New Jersey legislature in late March 2020 [3]. However, the financial and economic losses caused by the COVID-19 outbreak are so serious that the insurance industry cannot bear them alone [2]. If the financial and economic losses from the COVID-19 epidemic are transferred to the insurance and reinsurance industry, it will have a significant impact on their capital and solvency [2]. Only a government-funded reinsurance mechanism for infectious disease risks can protect the vulnerable small enterprises in the event of an epidemic and maintain the proper functioning of the insurance industry [2,4,5,6].

In China, due to the COVID-19 epidemic, China’s GDP fell 6.8% year-on-year in the first quarter of 2020, the first negative growth rate since 1992 and a new low growth rate in the 40 years of the implementation of reform and opening up since 1978 [4,5]. As an important member of the economy and society, the insurance mechanism participates in the specialization of division of labor in society and assumes the responsibility of managing risks and organizing loss compensation, thus determining that risk diversification and organizing loss compensation are the basic functions inherent in insurance [7]. The risk diversification function forms an insurance fund by collecting premiums to concentrate a large number of units in the same risk category, so that a small number of risk shocks will be covered [6]. The organizational loss compensation function financially compensates a small number of units that suffer losses due to risky incidents, and its insurance fund is established jointly by the majority [8].

In the COVID-19 epidemic, Chinese insurance companies failed to perform their essential functions effectively [4]. Although many insurance companies in China have responded positively by speeding up the claims process, shortening the waiting time for the COVID-19 patients, expanding insurance coverage, and actively donating funds to the infected areas, none of these actions indicates that insurance companies have performed their essential functional role [4]. The basic function of an insurance company should be judged by the insurance company’s payout costs and its ability to assume full responsibility for the compensation of losses. According to China’s Insurance Association as of 10 April 2020, Chinese insurance companies had paid out a total of RMB 347 million as a result of the COVID-19 epidemic, which does not exceed 0.2% of the economic loss. China’s insurance companies have failed to spread the risk and assume the responsibility for loss compensation effectively [4]. In addition, as of 10 April 2020, the data of the China Insurance Association showed that the accumulated social donations amounted to RMB 376 million, which was higher than the amount paid out by the insurance mechanism, reflecting the failure of the insurance mechanism to play its essential role in the COVID-19 epidemic effectively [4].

In summary, it can be seen that the role of the insurance mechanism in the early stages of the COVID-19 epidemic differed significantly between the U.S. and China [2,4]. How to play the role of insurance mechanism in infectious disease risk prevention effectively? What are the challenges of the insurance mechanism in infectious disease risk prevention? How to make an effective top-level design for infectious disease insurance? These are both the focal questions of academic concerns and the core questions discussed in this paper.

**Figure 1:**
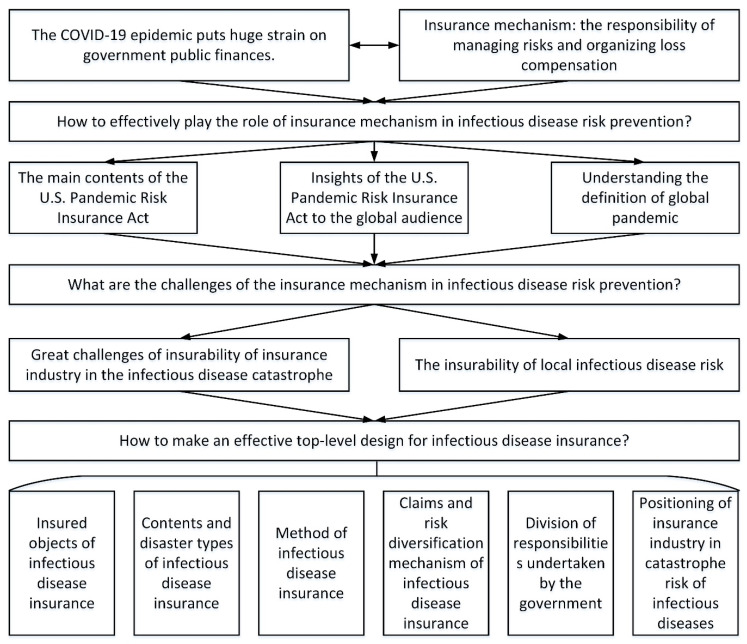
The logical framework of this paper.

To answer the above questions, this paper first analyzes the key elements of the U.S. Pandemic Risk Insurance Act to understand how the insurance mechanism works in the United States in the face of a pandemic, and then describes its implications for the global audiences. On this basis, this paper provides a definition of a global pandemic, an analysis of the challenges to the insurability of infectious disease catastrophes, and the insurability that localized infectious disease risks possess. Finally, this paper analyzes the top-level design of the risk transfer mechanism for infectious diseases in terms of coverage targets, coverage content, coverage catastrophes, the selection of insurance distribution channels, claims handling, the risk diversification mechanism, government responsibility, and the positioning and value of the insurance industry in the catastrophe risk of infectious diseases (Figure 1).

## 2. The Main Contents of the U.S. Pandemic Risk Insurance Act

In March 2020, the House Financial Services Committee of the U.S. called for the passage of the Pandemic Risk Insurance Act to provide reinsurance for the insurance industry in the event of a similar global pandemic in the future [5,6]. The U.S. Pandemic Risk Insurance Act refers to the design idea of the U.S. Terrorism Risk Insurance Program [2]. It is a non-compulsory project; the insurance companies can participate voluntarily. However, once participating in this project, the insurance companies shall not refuse to cover the risk of infectious diseases when the sales business is interrupted [2]. Insurers and reinsurers can obtain government-provided infectious disease reinsurance only if they pay reinsurance premiums to the government. The reinsurance premiums are transferred to the Pandemic Risk Reinsurance Fund, which is administered by the U.S. Treasury. Some global pandemic risks may be transferred to the capital market and international reinsurance market. The establishment of the U.S. Pandemic Risk Insurance Act can ensure that the insurance industry can provide a reasonable price of infectious diseases business interruption insurance and maintain social resilience. In the design of the U.S. Pandemic Risk Reinsurance Fund, the retained loss of the insurance industry is USD 250 billion, and the excess part is covered by the U.S. government, and the maximum national compensation is no more than USD 500 billion [2,3].

## 3. Insights of the U.S. Pandemic Risk Insurance Act to the Global Audiences

Given the initial outbreak of an infectious disease catastrophe, it cannot be known whether the government alone can fully cope with its adverse effects, especially in critical areas involving public health and economic development [4]. Due to the limited nature of the government’s public finances, there are two possible dilemmas for public finances [8].

The first dilemma is that government public finance has a budget for responding to a global pandemic, but limited public finance cannot quickly and effectively fund a global pandemic that affects a large number of people [5].

The second dilemma is that government public finance does not have a budget for responding to an infectious disease catastrophe. In the event of an infectious disease catastrophe in the context of interconnectedness, the government has the responsibility to respond in the first instance due to its highly contagious nature [6]. The government would have to break its original public finance budget, which would inevitably have an impact on the development of other sectors and may not be able to respond effectively to an infectious disease catastrophe [9].

Both dilemmas suggest that sudden-onset infectious disease catastrophes are difficult to respond to effectively by the government public finance alone due to their widespread impact, a phenomenon reflected in the response to the COVID-19 epidemic in many countries around the world [4,6]. Therefore, responding to infectious disease catastrophes requires reliance on market mechanisms.

Insurance mechanisms have the advantage of good capital financing and help relieve the pressure on the public finance of the government [10,11]. The Pandemic Risk Insurance Act discussed in the U.S. during the COVID-19 epidemic gives an initial way for different countries and regions around the world to respond to infectious disease catastrophes [2,3]. Through the effective combination of government public finance and the insurance mechanism, i.e., insurance companies provide infectious disease insurance to the public, and the government provides reinsurance coverage to insurance companies with the support of relevant policies, the pressure on government public finance can be relieved, and the smooth operation of insurance companies can be guaranteed. It is on the basis of understanding the global pandemic that this paper attempts to describe the relevant role of the insurance mechanism.

## 4. The Definition of a Global Pandemic

According to the World Health Organization, a global pandemic is a new disease with widely human-to-human transmission worldwide [2]. Usually, an infectious disease becomes a global pandemic when its occurrence becomes quite common in a particular country or many regions. [4]. As it spreads widely in many parts of the world, it becomes a global pandemic. A global pandemic infects more people, causes more deaths, and may have broad social and economic impacts [12]. In the past 100 years, there have been many kinds of global pandemic events in the world [13]. For example, the Spanish Influenza in 1918–1920 killed 20–50 million people all over the world [12]. The Asian Flu in 1957–1958 killed about 200 thousand people. HIV/AIDS has killed about 35 million people since 1981. Influenza A in 2009 killed about 284 thousand people. The Ebola Virus in 2014–2016 killed 11.3 thousand people all over the world [3].

## 5. Challenges of Insurability in the Infectious Disease Catastrophe

The fundamental reason that the U.S. government began discussing the creation of a government-sponsored Pandemic Risk Insurance Act is the enormous challenge of insurability for infectious disease catastrophes. Insurance companies are unable to provide sufficient underwriting capacity to the market [8,11,14]. Instead, it is necessary to establish a reinsurance mechanism for the infectious disease risk financed by the government [9,15,16].

Infectious disease catastrophes differ from natural disaster catastrophes in four dimensions, leading to significant challenges in their insurability: regional impact, risk accumulation, correlation with capital markets, and accuracy of catastrophe model [2].

In the dimension of regional impact, due to the rapid development of globalization characterized by interconnection [17], infectious disease catastrophes can spread rapidly throughout the country and even the world [18]. Natural disasters, whether typhoon, earthquake, or flood, are regional events, which are unlikely to have an impact on all regions of the world [10,19].

In the dimension of risk accumulation, there are quantitative differences in risk accumulation due to the region of influence [12]. Infectious disease catastrophes are global risk accumulations, which affect many types of insurance at the same time, such as life insurance, business interruption, activity cancellation insurance, credit guarantee insurance, etc. However, in general, there is only regional risk accumulation in natural disaster risks [20]. Insurance companies can control key scenarios, such as the East Japan earthquake in 2011, the American hurricane in 2005, etc., to ensure that even if a natural disaster catastrophe event occurs, it will not have an unbearable impact on the insurance companies’ solvency [8].

In the dimension of correlation with capital markets, the risk of serious infectious diseases has a high correlation with capital markets [21]. For example, the COVID-19 epidemic led to multiple fusions of the U.S. stocks; the global stock market plummeted, and the economy entered recession [2]. However, the correlation between natural disaster catastrophe risks and capital markets is relatively low, which is why many investors are willing to diversify the systemic risk in their portfolios by purchasing catastrophe bonds [22,23,24].

In the dimension of the accuracy of the catastrophe model, if insurance companies cannot carry out actuarial pricing for a risk, they will lose the basis of underwriting. For the infectious disease catastrophe risks, the loss of the insurance industry is greatly affected by human factors [3].

Different measures taken by the government (e.g., disrupting transportation, closing factories, etc.) may have very different and unpredictable results for controlling the spread of infectious diseases [4]. However, for natural disaster risks, although they are also highly episodic, the return periods and corresponding loss of natural disaster risks can be quantified and analyzed by summarizing historical events, constructing stochastic event sets, and building catastrophe risk models [4,9,25].

## 6. Understanding the Insurability of Local Infectious Disease Risk

In the traditional Chinese property insurance business interruption clause, there are some specific requirements for the infectious disease extension clause [25]. Firstly, the infectious diseases must occur at the place of business; if the infectious diseases occur only in the immediate area of the place of business, it does not meet the trigger conditions. Secondly, the business premises must be closed by the government, and if the business premises are closed only because of fear of an infectious disease, the trigger is not met. Thirdly, the outbreak of the infectious disease results in a loss of profits due to the interruption of business [26,27,28]. The purpose of this infectious disease extension is to protect against some local infectious diseases, such as Legionella or Salmonella. The former is usually contracted through inhalation of water spray from contaminated water sources, while the latter is caused by consumption of contaminated food. [2,3].

## 7. Key Points of Insurance Top-Level Design of Infectious Disease risk Transfer Mechanism in China

**Figure 2:**
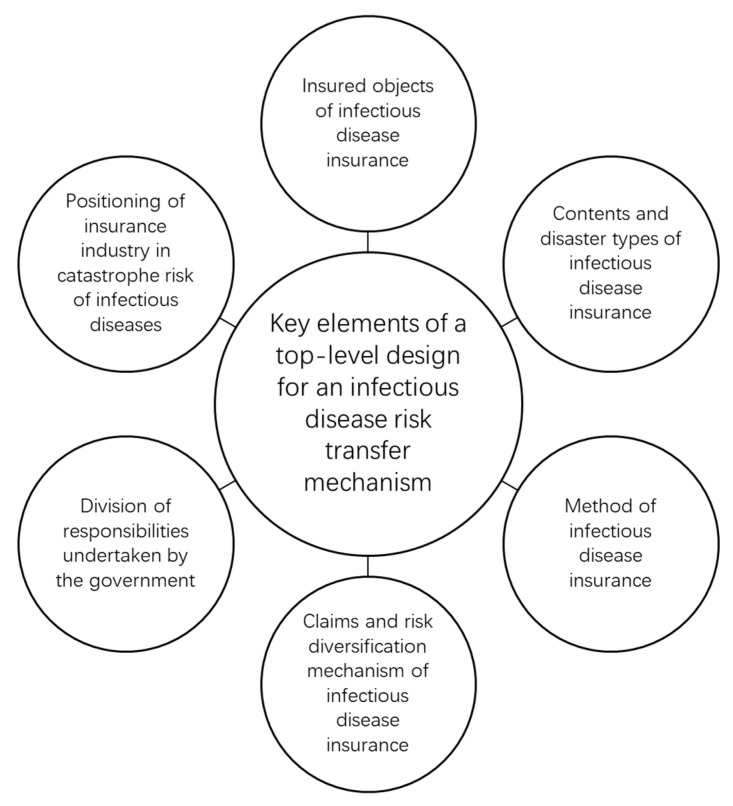
Top-level design of infectious disease risk transfer mechanism.

It is necessary for the government to take the lead to establish the infectious disease risk transfer mechanism financed by the government, so as to provide reinsurance to the insurance market [29,30,31,32]. The following key issues need to be considered in the top-level design of the infectious disease risk transfer mechanism in China (Figure 2).

### 7.1. Insured Objects of Infectious Disease Insurance

The international discussion on the establishment of a catastrophe insurance system for infectious diseases mainly focuses on how to provide insurance for small- and medium-sized enterprises [9]. The reason is that small- and medium-sized enterprises have a great impact on employment and social stability. For example, small- and medium-sized enterprises contribute more than 60% of the GDP and more than 80% of jobs in China [4]. However, when COVID-19 happened, small- and medium-sized enterprises in catering, tourism, retail, and other fields were greatly impacted. Many of them were at risk of having their capital chains broken, and they desperately needed help from contagious disease insurance. [3].

As for the large enterprises related to people’s livelihood, if they encounter problems, the government will also consider the social impact and decide whether to carry out rescue or not [12]. After the occurrence of the COVID-19 epidemic, the contradiction between public finance revenue and expenditure of local governments in China is prominent [4]. On the one hand, public spending increased due to the control of the COVID-19 prevalence and stimulation of consumption [2]. On the other hand, tax revenue was significantly reduced due to economic downturn and tax-free measures.

However, considering the risk accumulation of a global epidemic, the insurance industry does not have sufficient unwind capacity to absorb the public financial risk of local government [3]. The contradiction between local government’s public revenue and expenditure mainly depends on issuing bonds and transferring payment from central government [33,34].

### 7.2. Contents and Disaster Types of Infectious Disease Insurance

It can be seen from the COVID-19 epidemic that the economic losses of enterprises in various industries in China come from the following four aspects. The first aspect is the direct economic losses caused by business interruption caused by infectious diseases, such as tourism, education, etc. [4]. The second aspect is people’s psyche and pandemic anxiety [2]. The third aspect is the loss of profits caused by the disruption of the upstream and downstream supply chain caused by infectious diseases, such as the shortage of raw materials in some manufacturing industries [3]. The fourth aspect is the profit loss caused by the shrinking market demand caused by infectious diseases, such as the sharp decrease in orders faced by foreign trade enterprises [4].

When the cause of profit loss changes from direct to indirect, it is more difficult to settle the loss [35]. When designing the insurance content of infectious disease insurance, in principle, it should be that the government’s limited public financial funds should be provided to the most needed enterprises to ensure the stability of social and economic order [36,37].

It is the core issue of the risk transfer mechanism of infectious diseases to protect only global epidemics or all infectious diseases. If the design of infectious disease catastrophe insurance is limited only to cover the global pandemic, the trigger frequency can be effectively reduced. However, considering that most of the insured cannot distinguish between a global pandemic and a general infectious disease, it is very easy to have disputes when settling claims [38,39]. If all infectious diseases are included in the insurance design, it is easier for the insured to understand, but such risk will be triggered more frequently, so a higher rate support is needed [40,41,42].

### 7.3. Method of Infectious Disease Insurance

Generally, the sales mode of insurance products can be divided into three types: compulsory insurance, semi-compulsory insurance, and voluntary insurance [8]. Among many catastrophe insurance projects in the world, the three kinds of insurance sales modes are very common, and countries need to choose their own according to their national conditions.

To require all enterprises to purchase compulsory insurance can ensure the coverage of infectious disease insurance, which cannot only quickly accumulate premium in normal years, but also ensure that most enterprises can get compensation in case of a pandemic. However, compulsory insurance will increase the operating costs of enterprises, which may be resisted by enterprises.

The semi-compulsory insurance means that once the enterprise has purchased the profit loss insurance, it will be forced to extend the infectious disease protection. Infectious disease insurance can also be extended to all property insurance policies. At present, the coverage rate of enterprise profit loss insurance is very low, while the coverage rate of enterprise property insurance is high for large enterprises and low for small- and medium-sized enterprises. Therefore, the coverage of semi-compulsory insurance for small- and medium-sized enterprises is very limited.

The form of voluntary insurance fully respects the needs of enterprises, but there will be adverse selection problems. At the same time, if the coverage rate is too low, it will not achieve the original function of the infectious disease insurance.

### 7.4. Claims and Risk Diversification Mechanism of Infectious Disease Insurance

Once a global pandemic occurs, there will be countless insured reporting cases in a short period of time, which is very similar to the situation after a natural disaster catastrophe. Therefore, it is not recommended to adopt the traditional operation mode of business interruption insurance for large enterprises. Instead, online and electronic methods should be adopted as far as possible, and artificial intelligence technology should be used to simplify the claim settlement process. Through the prepayment of compensation to solve the problem of enterprise liquidity, it is possible to determine the final amount of compensation after the detailed loss.

The decentralized mechanism of infectious disease risk can be divided into two types: stratified sharing and government full bearing [9]. The reference cases of stratified sharing include the earthquake insurance system of Japan and the terrorism risk insurance act of the U.S. The top layer of the risk dispersion mechanism is provided by the government through public finance and tax funds; the middle level is borne by insurance companies and the capital market; and the low level is the risk of some frequent small infectious disease events covered by insurance companies themselves. The reference cases of government full bearing include the flood insurance system of the U.S., the earthquake insurance system of New Zealand, and the catastrophe insurance fund of Turkey [11]. In this kind of risk dispersion mechanism, the government bears all the risks, while the insurance company only undertakes the compensation, loss determination, distribution, and value-added services.

### 7.5. Division of Responsibilities Undertaken by the Government

In the reinsurance scheme of infectious diseases, the government has two choices: limited responsibilities and unlimited responsibilities.

In the government’s limited responsibilities, the catastrophe fund has agreed on the compensation limit in advance, and the government bears the limited responsibilities [10]. The advantage of the government’s limited responsibilities is to avoid too much impact on the government’s public finance fund, but the disadvantage is that the compensation limit may be exceeded in the event of a catastrophe. In this case, it is necessary to determine the distribution mode of limited funds in advance. However, no matter how the government’s public finance fund is allocated, some of the insured may not be able to get full compensation. If it happens, the government may decide to add funds temporarily to avoid causing dissatisfaction among the insured [8].

In the government’s unlimited responsibilities, the compensation limit is not capped. The advantage of the government’s unlimited responsibilities is that the government has unlimited coverage of insurance responsibilities, which enhances market confidence, while the disadvantage is that the government has too much uncertainty in predicting the amount of compensation for catastrophes.

### 7.6. Positioning of Insurance Industry in Catastrophe Risk of Infectious Diseases

Because the global pandemic is not insurable, the value of the insurance industry is not reflected in the underwriting capacity, but should be reflected in some value-added services. For example: implement risk management in advance; help the insured to prevent disasters and reduce losses in the first time when a health and safety incident occurs; accurately release compensation through compensation and loss determination after the event; promote the insured to incorporate risk management into daily operation through risk pricing.

## 8. Discussion

In China, the insurance mechanism has failed in response to the COVID-19 epidemic [4]. The reasons for the failure of the insurance mechanism in China to play an effective role in the COVID-19 epidemic include the following four main aspects.

Firstly, the development of China’s insurance industry is still in its infancy, and the low coverage rate of commercial insurance is the fundamental reason why insurance failed to play its basic function effectively in the COVID-19 epidemic.

Secondly, the small variety of insurance products and limited risk coverage in China are the direct reasons why insurance companies failed to play their basic functional role in the COVID-19 epidemic effectively.

Thirdly, China has a large gap in catastrophe protection, and the proportion of losses borne by insurance is small. In countries with high insurance penetration, the proportion of insurance compensation for catastrophic economic losses reaches about 50%, and, in some developed countries, the proportion of compensation for catastrophic losses even reaches 60–70%, but in China the proportion of compensation is only less than 5% [4].

Fourthly, the boundary between the role of China’s insurance market and government is unclear; the role of insurance for catastrophes and serious public emergencies is unclear; and the development of the industry is limited.

In order to solve the dilemmas of China’s insurance mechanism in infectious disease catastrophe response, the following four aspects may need to be improved.

Firstly, it is necessary to improve the catastrophe insurance market in China and increase the insurance coverage. To improve China’s catastrophe insurance market, the premise is to establish a hierarchical catastrophe insurance system in line with China’s national conditions, with policy insurance providing basic protection and commercial insurance as a supplement, while introducing reinsurance for risk redistribution. In addition, to achieve wide coverage of catastrophe insurance, the government should focus on the education and publicity of residents on catastrophe insurance to improve the public’s awareness of risk prevention.

Secondly, it is necessary to encourage innovation of insurance products and increase the risk coverage. First, a product innovation protection mechanism can be established at the legislative level, and, at the same time, appropriate financial subsidies can be provided to insurance companies for their research and development expenses to mobilize their enthusiasm for innovation. Second, insurance professionals should be cultivated and encouraged to participate in the design and innovation of insurance products. Furthermore, the innovation of insurance products should consider the differentiated geographical factors and make use of diversified insurance products to increase the scope of insurance liability and risk coverage according to local conditions. The government firmly resists the act of using insurance product innovation as a gimmick for hype and deviating from the sound business requirements and social responsibilities of insurance companies. In addition, the market competition can be encouraged, so that competition can promote market product innovation, and product competition can achieve wide risk coverage.

Thirdly, it is necessary to strengthen the application of science and technology in the field of insurance to help the development of the insurance market. Attaching technology to the insurance market will bring many conveniences and opportunities to the insurance market in terms of pricing, underwriting, loss prevention, loss determination, claims, and other multiple links. On the one hand, technology can be applied to help insurance product innovation. For example, it is possible to establish catastrophe big data information management to optimize product pricing, and even use technology to assess the size of risk and differentiate pricing for regions and individuals accurately; its also possible to use technology to discover hidden risks and innovate products for improving insurance risk coverage. On the other hand, technology is applied to strengthen risk management and risk assessment. For example, technology, such as the Internet of Things, big data, artificial intelligence, etc., can be used to achieve scientific management and dynamic early warning of risks and effectively reduce the possible losses caused by catastrophe risks, and even block chain technology can be used to manage risks and reduce insurance moral hazard and insurance fraud. In general, applying technology to the insurance market can bring more possibilities to China’s insurance market.

Fourthly, it is necessary to coordinate the roles of government and market to improve the efficiency of market operation. Uncertainty in the boundary of the roles of government and market may constrain the development of the insurance industry, and constrain the coordination of the functional roles of both to maximize the operational efficiency of the insurance market. In the insurance market, the main role of the government should be to regulate the market operation and create a good business environment. To this end, the government can introduce and improve relevant laws and regulations to make the insurance market lawful.

## 9. Conclusions

This paper describes the role of the insurance mechanism in responding to pandemics. This paper introduces the main contents and insights of the U.S. Pandemic Risk Insurance Act and defines the basic concept of global pandemics. The paper describes the challenges faced by the insurance mechanism in responding to global infectious disease catastrophes in four dimensions: region of impact, risk accumulation, relevance to capital markets, accuracy of catastrophe models, and the insurability of localized infectious disease risks. Finally, this paper elaborates on the top-level design of the risk transfer mechanism for infectious diseases from the aspects of insured targets, coverage contents, coverage types, selection of insurance distribution channels, claims handling, risk dispersion mechanism, governmental commitment, and the positioning and value of the insurance industry in the catastrophic risk of infectious diseases. Through the above, this paper argues that the insurance mechanism is necessary to cope with infectious disease risk, and the key lies in the scientific design of the insurance mechanism for infectious disease risk.

## Data Availability

No new data were created or analyzed in this study. Data sharing is not applicable to this article.

## References

[1] International Monetary Fund (2020). World Economic Outlook, October 2020: A Long and Difficult Ascent.

[2] International Monetary Fund (2020). World Economic Outlook Update, June 2020: A Crisis Like No Other, An Uncertain Recovery.

[3] International Monetary Fund (2020). World Economic Outlook, April 2020: The Great Lockdown.

[4] State Council of China Review of the Authoritative Release of the Joint Prevention and Control Mechanism of the State Council. http://sousuo.gov.cn/column/49276/0.htm.

[5] Kong F. (2020). Understanding and strengthening the role of catastrophe lottery in catastrophe risk transfer system. J. Contingencies Crisis Manag..

[6] Kong F. (2020). Better understanding positive and negative impacts of disasters on regional economies, with special reference to China. J. Contingencies Crisis Manag..

[7] Kong F., Sun S. (2021). Better understanding insurance mechanism in dealing with climate change risk, with special reference to China. Int. J. Environ. Res. Public Health.

[8] Li M. (2016). Study on Raising social Fund to Redistribute Disaster Risk with Lottery and Insurance. Ph.D. Thesis.

[9] Shi P., Li M. (2014). New model of catastrophe risk transfer. China Financ..

[10] Shi P., Jeager C., Ye Q. (2013). Integrated Risk Governance.

[11] Shi P. (2019). IHDP/Future Earth-Integrated Risk Governance Project Series: Disaster Risk Science.

[12] World Economic Forum (2020). Global Risk Report 2020.

[13] Lv X., Xue L. (2016). Managing the unexpected: Sense-making in the Chinese emergency management system. Public Adm..

[14] Shi P., Xu W., Wang J. (2016). Natural Disaster System in China. Natural Disasters in China.

[15] Kong F., Wang Y., Lv L., Meng Y., Shi P. (2018). Progress and prospect of the global and complex impact of catastrophe on economy in the context of interconnection. J. Cent. China Norm. Univ..

[16] Surminski S. (2014). The role of insurance in reducing direct risk: The case of flood insurance. Int. Rev. Environ. Res. Econom..

[17] Walker G.R., Mason M.S., Crompton R.P., Musulin R.T. (2016). Application of insurance modelling tools to climate change adaptation decision-making relating to the built environment. Struct. Infrast. Engine..

[18] IPCC Climate Change (2014). Contribution of Working Groups I, II and III to the 5th Assessment Report of the Intergovernmental Panel on Climate Change.

[19] Nam S.W. (2017). A study on the effects and adaptation of climate change in insurance industry. J. Clim. Chang. Res..

[20] Stahel W.R. (2009). In favour of a proactive insurance approach to climate change. Geneva Pap. Risk Insur. Issues Pract..

[21] Gesualdo G., Souza F., Mendiondo E.M. (2020). Insurance Fund as an Adaptation Measure for Increasing Water Security in Basins under Change.

[22] Meng Y., Yang S., Shi P., Jeager C.C. (2015). The asymmetric impact of natural disasters on China’s bilateral trade. Nat. Hazards Earth Syst. Sci..

[23] Valente D., Miglietta P.P., Porrini D., Pasimeni M.R., Zurlini G., Petrosillo I. (2019). A first analysis on the need to integrate ecological aspects into financial insurance. Ecolog. Model..

[24] Porrini D., Schwarze R. (2014). Insurance models and European climate change policies: An assessment. Eur. J. Law Econom..

[25] Candel F.M. (2007). Climate change and the global insurance industry: Impacts and problems in Latin America. Geneva Pap. Risk Insur. Issues Pract..

[26] United Nations International Strategy for Disaster Reduction (2019). Global Assessment Report on Disaster Risk Reduction 2019.

[27] United Nations International Strategy for Disaster Reduction (2015). Sendai Framework for Disaster Risk Reduction 2015–2030.

[28] United Nations International Strategy for Disaster Reduction (2013). Global Assessment Report on Disaster Risk Reduction 2013.

[29] Cremades R., Surminski S., Máez Costa M., Hudson P., Shrivastava P., Gascoigne J. (2018). Using the adaptive cycle in climate-risk insurance to design resilient futures. Nat. Clim. Chang..

[30] Crichton D. (2002). UK and global insurance responses to flood hazard. Water Int..

[31] Cutter S.L. (2016). Resilience to what? Resilience for whom?. Geogr. J..

[32] Dlugolecki A.F. (2000). Climate change and the insurance industry. Geneva Pap. Risk Insur. Issues Pract..

[33] Eisenack K., Stecker R., Reckien D., Hoffmann E. (2012). Adaptation to climate change in the transport sector: A review of actions and actors. Mitiga. Adapt. Strateg. Glob. Chang..

[34] Fleming D., Noy I., Pastor P.J., Owen S. (2018). Public insurance and climate change (part one): Past trends in weather-related insurance in New Zealand. Work. Pap..

[35] Florence C., Katie J., Swenja S. (2016). Strengthening insurance partnerships in the face of climate change—Insights from an agent-based model of flood insurance in the UK. Sci. Total Environ..

[36] Gloor M., Holzheu T., Tamm K. (2020). Modelling climate change risk for the insurance industry. Occas. Pap. Case Stud. Environ. Risk Anal. Methodol..

[37] He Q. (2016). Mitigation of climate change risks and regulation by insurance: A feasible proposal for China. Boston Coll. Environ. Aff. Law Rev..

[38] Michael H. (2007). Climate change and the global insurance industry. Geneva Pap. Risk Insur. Issues Pract..

[39] Jrgensen S.L., Termansen M., Pascual U. (2020). Natural insurance as condition for market insurance: Climate change adaptation in agriculture. Ecol. Econ..

[40] Jangle N., Mehra M., Dror D. (2016). Climate cost of cultivation: A new crop index method to quantify farmers’ cost of climate change exemplified in rural India. Geneva Pap. Risk Insur. Issues Pract..

[41] Leblois A., Cotty T.L., D’Htel E.M. (2020). How might climate change influence farmers’ demand for index-based insurance?. Ecol. Econ..

[42] Mills E. (2009). A global review of insurance industry responses to climate change. Geneva Pap. Risk Insur. Issues Pract..

